# Ocular Jarisch-Herxheimer Reaction in the Treatment of Ocular Syphilis: A Case Report and Review of the Literature

**DOI:** 10.7759/cureus.33696

**Published:** 2023-01-12

**Authors:** Noranida Abd Manan, Azlan Azha Musa, Pooi Wah Lott, Mimiwati Zahari, Iqbal Tajunisah

**Affiliations:** 1 Department of Ophthalmology, Universiti Malaya Eye Research Centre, University of Malaya, Kuala Lumpur, MYS; 2 Department of Ophthalmology, Sungai Buloh, Universiti Teknologi MARA, Selangor, MYS

**Keywords:** jarisch-herxheimer, syphilis uveitis, ocular syphilis, macular edema, macular atrophy

## Abstract

Jarisch-Herxheimer reaction (JHR) is a transient clinical phenomenon in patients with syphilis who receive antibiotic treatment. A 31-year-old man with an underlying HIV infection presented with worsening vision in the right eye two days after being treated with oral doxycycline for presumed left-eye neuroretinitis. Prior history revealed two episodes of penile discharge and ulcers that were not investigated. Examination showed bilateral optic disc swelling with right eye placoid chorioretinitis around the macula. Optical coherence tomography (OCT) demonstrated right macular edema and left macular thinning. Blood investigations confirmed syphilis infection. Subsequently, the patient was scheduled for a contrasted brain CT with oral steroid coverage due to underlying allergies. His vision incidentally improved soon after the short course of steroids. Repeated OCT demonstrated marked improvement of right macular edema, which we believe was secondary to JHR initiated by the earlier doxycycline treatment. Following oral steroid addition, improvement in vision and ocular findings were seen. At six-month post-treatment, there was right macular atrophy as a sequela of the macular edema. Ophthalmologists should be aware of ocular-related JHR complications, particularly in potentiating macular atrophy following macular edema upon initiating antibiotic treatment in syphilitic disease.

## Introduction

Ocular syphilis is a rare clinical manifestation of syphilis, affecting approximately 2.5-5% of patients with a confirmed diagnosis of syphilis [1]. Jarisch-Herxheimer reaction (JHR) is a transient clinical phenomenon that occurs in 10-25% of patients with syphilis who undergo antibiotic treatment [2]. JHR is usually characterized by systemic manifestations such as fever, chills, rigors, headache, myalgia, tachycardia, and hyperventilation and is rarely seen as an ocular complication. We described a case of ocular syphilis who developed worsening ocular symptoms after the commencement of oral doxycycline. We postulated that the condition was attributed to JHR.

## Case presentation

**Figure 1:**
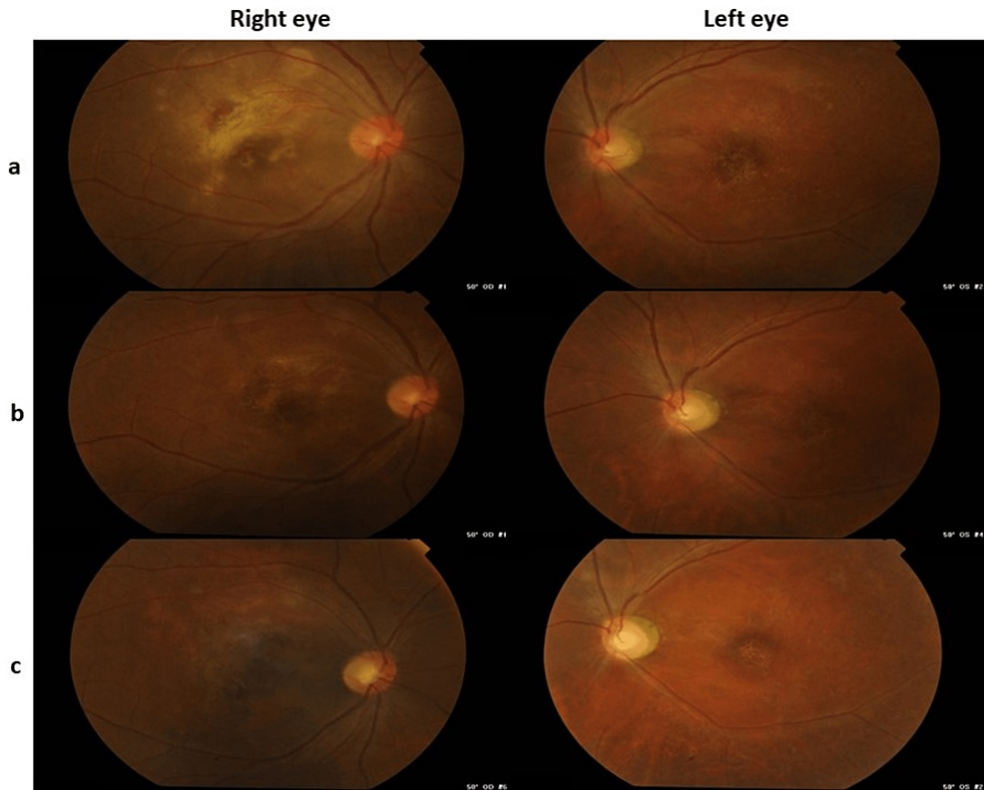
Color fundus photographs of both eyes at (a) initial presentation; (b) one month; and (c) six months after the initial presentation.

A 31-year-old man with premorbid good visual acuity in both eyes was referred by a private ophthalmologist for deterioration of the right eye visual acuity upon initiation of oral doxycycline for presumed left eye neuroretinitis. He initially presented to his ophthalmologist with five days history of left eye acute painless visual deterioration. However, two days following treatment, the fellow eye became blurry. Thus, the patient came to us for a second opinion. He also developed a low-grade fever, myalgia, and malaise a few hours after starting the antibiotic. The patient had a history of HIV infection diagnosed five years ago. He was not started on the highly active antiretroviral therapy (HAART) as his CD4+ count had not reached the commencement threshold. He admitted to having unprotected sex with multiple male partners in the past and had been in a monogamous relationship for the past two years. Prior history revealed two episodes of penile discharge and painless ulcers that were not investigated. He denied any history of drug or substance abuse. On examination, the best corrected visual acuity (BCVA) was 3/60 for the right eye and 2/60 for the left eye. There was a positive left eye relative afferent pupillary defect. The light-near dissociation was negative. Mild vitritis was present in both eyes. Dilated fundus examination (Figure 1a) of the right eye revealed a swollen and hyperemic optic disc with a large area of placoid chorioretinitis (two optic disc diameter) at the macula surrounded by smaller patchy areas of chorioretinitis. The left eye optic disc was hyperemic and swollen nasally with areas of pallor temporally. The macula appeared thinned out. Both eyes showed normal retinal vessels without vasculitis and hemorrhages.

**Figure 2:**
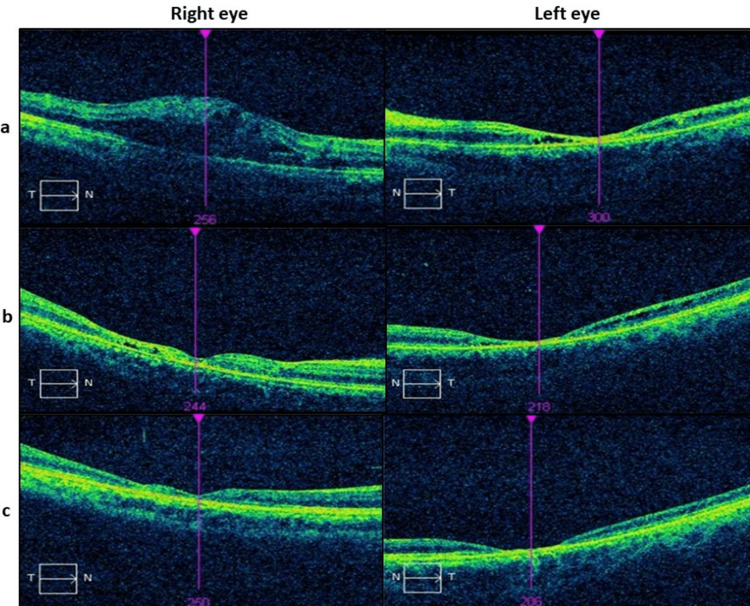
SD-OCT images of the macula of both eyes at (a) initial presentation; (b) one month; and (c) six months after the initial presentation. SD-OCT: Spectral-domain optical coherence tomography.

Spectral-domain optical coherence tomography (SD-OCT) (Figure 2a) of the right eye showed intraretinal cystic fluid with a central subfield thickness (CST) of 464 µm. In contrast, the left eye demonstrated a reduced CST of 74 µm with areas of cystic changes within the macula. In addition, OCT of the optic nerve showed a bilateral increase in retinal nerve fiber layer thickness with an average of 141 µm for the right and 109 µm for the left eye.

Based on the history of penile ulcers and discharge, a provisional diagnosis of bilateral ocular syphilis was suspected. Blood investigations revealed a non-reactive venereal disease research laboratory (VDRL) test but a positive Treponema pallidum particle agglutination (TPPA) test, which was repeated twice. His CD4+ cell count was moderately low at 288 cells/µL with a high HIV viral load value of 11,230 copies/mL. The erythrocyte sedimentation rate was high at 49 mm/hour. Other blood tests were within normal range, including full blood count, renal profile, and liver function. The Mantoux test and toxoplasma serology were negative, and the chest X-ray showed unremarkable findings. The patient was referred to the Infectious Disease (ID) team and subsequently was admitted for lumbar puncture and brain CT one week after his presentation. In the meantime, the prior treatment with oral doxycycline at 100 mg twice daily was continued while waiting for the syphilis results. As he had a known allergy, the patient was covered with oral prednisolone of 50 mg for three doses at twelve, seven, and an hour before the contrasted brain CT according to the hospital protocol. Interestingly, the patient noticed that his vision, especially the right eye, improved soon after, even before the standard treatment of syphilis. Brain CT was normal, and lumbar puncture tested negative for VDRL with a marginally raised protein level at 0.43 g/L. Other microbiological and biochemical tests were unremarkable.

At this juncture, his BCVA improved slightly to 6/60 in both eyes. Funduscopic examination (Figure 1b) revealed reduced optic disc swelling in both eyes with resolving areas of the previous chorioretinitis. SD-OCT (Figure 2b) demonstrated marked improvement in right macular cystic edema.

Since there were some clinical improvements and visual function soon after the commencement of systemic steroids, it was concluded that the patient developed a JHR triggered by the oral doxycycline prescribed earlier. The JHR was believed to have induced right macular cystic edema, further deteriorating the visual acuity. Initially, the decision by the ID team was to start him on a two-week course of IV penicillin G. However, he refused the associated prolonged hospital stay. He opted instead to be treated as latent syphilis. This decision was based on an overall negative cerebrospinal fluid (CSF) sample and a non-reactive serum VDRL with a positive TPPA on two separate occasions. He received intramuscular (IM) benzathine benzylpenicillin G 2.4 million units on day 14 of his presentation, then weekly for three weeks. He was also started on HAART consisting of 600 mg of efavirenz, 200 mg of emtricitabine, and 300 mg of tenofovir disoproxil fumarate once daily as well as a half-dose of oral prednisolone 30 mg daily throughout the antibiotic regime with gradual tapering within two weeks. No paradoxical worsening of his condition was observed upon receiving the IM antibiotic.

Six months after the initial presentation, the BCVA in both eyes slightly improved to 6/45 for distance and N48 for near vision. Funduscopic examination (Figure 1c) of the right eye showed an extensive area of macular atrophy with underlying scarring and crystalline deposits. The optic disc was pale temporally with the resolution of swelling. The left eye also had macular atrophy with crystalline deposits, but it was less widespread. In addition, the generalized pallor of the optic disc was observed. However, there was no improvement in macular thickness in both eyes, as seen in the SD-OCT (Figure 2c).

We referred him to the low-vision clinic and the Malaysian Association for the Blind for support. Currently, he is managing well with his preserved vision.

## Discussion

Ocular syphilis may be the only presenting symptom of a syphilitic infection. It is more common and severe in patients with HIV co-infection with a low CD4+ count [3, 4]. Among the many ocular manifestations of syphilis, uveitis remains the most common and can occur at any stage of syphilis [4, 5]. It has been established that syphilitic uveitis can occur as anterior, posterior, or keratouveitis [3]. In posterior uveitis, patients may have vitritis, retinitis, choroiditis, vasculitis, retinal hemorrhages, optic disc swelling, neuroretinitis, and exudative retinal detachments [6]. The CDC guidelines in 2015 recommended that ocular syphilis be treated as neurosyphilis.

Our case illustrates an unusual form of bilateral involvement of the posterior pole in syphilitic uveitis. This patient's low CD4+ count was the reason for the poor immune response to mount for the more typical presentation of vitritis, vasculitis, and anterior uveitis seen in an immunocompetent person with ocular syphilis. The diagnosis of syphilis largely depends on serology, where both non-treponemal and treponemal tests are used in tandem to ensure a sensitive and specific diagnosis [4, 5]. In our patient, a reverse-sequence algorithm was used to diagnose syphilis [7]. The TPPA (treponemal test) was positive initially, but VDRL (non-treponemal test) was negative. Further positive TPPA tests performed on a separate occasion confirmed the diagnosis of syphilis for this patient. Non-treponemal tests in an HIV-infected person can be falsely negative in early syphilis due to the prozone phenomenon or in severely immunocompromised individuals [8].

JHR is proposed to be an exaggerated systemic response towards the release of treponemal antigens from the dead spirochetes following the initial antibiotic regime. JHR is a clinical diagnosis mainly, and it is often unrecognized and under-reported. Symptoms may include malaise, pyrexia, muscle ache, and worsening of the ocular complications of syphilis [9]. Ocular manifestations of JHR are rare, with only a few cases reported previously. We performed a comprehensive search for articles and case reports describing ocular manifestations of JHR using MEDLINE. Using the terms "ocular," "intraocular," and "Jarisch-Herxheimer reaction," we found 14 articles. After reviewing these articles and their bibliographies, six articles were included in the literature review (Table 1). Non-English Language articles were excluded.

**Table 1:** Previous literature on ocular manifestations of JHR. VA: Visual acuity: OD: Oculus dexter; OS: Oculus sinister; OU: Oculus uterque; ATT: Anti-tuberculous therapy; TB: Tuberculosis; HM: Hand movement; JHR: Jarisch-Herxheimer reaction.

Case report	Underlying disease and organism	Onset of symptoms	Ocular symptoms	Systemic involvement	VA at onset	Ocular findings	Treatment	Final VA
Ramtohul et al., 2018 [10]	Lemierre syndrome, Fusobacterium necrophorum	A few days after parenteral piperacillin-tazobactam	Blurred vision	None	OU 20/32	Bilateral cystoid macular edema with mild peripheral retinal vasculitis, diffuse and segmental perivascular sheathing	Single intravitreal injection of Ranibizumab 0.5 mg (0.05 mL) in both eyes	OU 20/20
Siantar et al., 2015 [11]	Panuveitis with tuberculous chorioretinitis OS, Mycobacterium tuberculosis	3 months after oral ATT	Blurred vision	None	OS HM	Vitritis, vasculitis and chorioretinal lesions	Oral prednisolone 40 mg once daily	OS 6/120
Neunhöffer et al., 2014 [12]	Latent TB, Mycobacterium tuberculosis	3 days after oral isoniazid	Blurred vision	None	OU 20/200	Mild anterior chamber reaction, optic disc swelling, cystoid macular edema and periphlebitis	Methylprednisolone 0.5 mg/kg to 1.0 mg/kg, gradually tapered over 6 weeks	OU 20/25
Cheung et al., 2009 [13]	TB OS chorioretinitis, Mycobacterium tuberculosis	4 days after oral ATT	Blurred vision	None	OS 6/120	Moderate anterior chamber reaction, vitritis and retinitis with perivascular sheathing	Oral prednisolone 25 mg once daily, gradually tapered over 1 month	OS 6/45
Fathilah et al., 2003 [9]	Ocular syphilis, *Treponema pallidum*	6 hours after intravenous penicillin	Blurred vision	Fever, chills and rigors, intense headache, generalized pruritus	OD 4/60 OS 1/60	Moderate anterior chamber reaction, vitritis, swollen and hyperemic disc, macula edema and macula star	Oral prednisolone 60 mg once daily for two weeks, then gradually tapered	OD 6/12 OS 6/60
Playford et al., 1992 [14]	Whipple's disease, Corynebacterium jeikeium	12 hours after oral co-trimoxazole and intramuscular streptomycin	Blurred vision	Fever, confusion	OU 6/12	Bilateral multiple retinal vasculitis	Oral prednisolone 30 mg once daily, gradually tapered over 2 months	OD 6/9 OS 6/6

Ocular manifestations of JHR were not only seen in patients with syphilis, but it has also been observed in other bacterial infections such as Whipple's disease [14], tuberculosis (TB) [11-13], and Lemierre syndrome [10]. The latest four cases reported were of isolated ocular JHR [10-13]. In these case reports, patients experienced deterioration of vision either in one eye or bilaterally at the onset of JHR. The onset of ocular JHR was unpredictable. It varied from as early as six hours following the initiation of treatment up to three months later. Generally, the ocular findings included uveitis, vitritis, optic disc swelling, macular edema, retinal vasculitis, and neuroretinitis. There were no methods advocated to prevent JHR except to warn patients of this occurrence and to provide supportive care in generalized JHR, such as monitoring vital signs and administering fluids, antipyretics, and analgesics because such reactions will usually subside within 24-36 hours [15]. Most ophthalmologists have used a short course of corticosteroids to lessen the effects of ocular JHR in their patients, with relatively better visual outcomes. Nevertheless, no standardized dose and duration of corticosteroid therapy was recommended. Alternatively, Ramtohul P et al. reported the usage of intravitreal ranibizumab 0.5 mg in their patient due to underlying severe unstable hypertension [10]. Their patient showed significant visual improvement within one month after a single intravitreal injection, with no recurrent central macular edema or retinal vasculitis after a four-year follow-up. Intravitreal injection of ranibizumab could be an option when systemic steroid therapy is contraindicated, such as in patients with uncontrolled diabetes, active or latent infections, severe hypertension, and unstable mental disorders [10].

Both ocular syphilis and JHR can result in a macular lesion. Acute posterior placoid chorioretinitis lesions in ocular syphilis can be recognized from the macular OCT by loss of ellipsoid zone at the fovea, perifoveal thickening of photoreceptors, hyperreflectivity of the choroid at the fovea, with or without cystoid degeneration [16]. In contrast, macular lesions in ocular JHR are generally confined to the presence of cystoid macula edema, as described by three cases reported previously [9, 10, 12]. In our case, the patient only noticed a deterioration of his right eye's visual acuity after 48 hours of initial doxycycline therapy with resolution of the macular edema after systemic steroid therapy. Therefore, we postulated that the macular edema that occurred in our patient was more suggestive of the ocular JHR. Macular edema, in this case, is likely a result of the disruption of the inner blood-retinal barrier from the inflammatory process of the JHR. Our patient subsequently suffered from macular atrophy, which is not an uncommon complication seen in both ocular syphilis and JHR [11, 17]. It has been shown that healed chorioretinitis in ocular syphilis can manifest with nonspecific findings, including choroidal and retinal atrophy, due to underlying ischemia [18]. The extensive outer retinal cell loss of the left eye at the initial presentation could also contribute to the macula scarring. On the other hand, the right macular atrophy was further compounded by the cystic macular edema, which we believed occurred from the JHR. The subsequent resolved macular edema with steroid therapy contributed to the development of worsened macula thinning and atrophy. Macular atrophy and fibrosis developed over time after the acute event due to the permanent damage to macular retinal cells leading to substantial visual impairment in our patient [19].

## Conclusions

Our case portrayed an unusual incidence of macular atrophy as a sequela of a treated case of ocular syphilis, resulting in poor visual outcomes. We believed that there were elements of JHR that further accelerated and worsened the condition. Ophthalmologists need to be aware of ocular JHR in managing syphilitic eye disease to facilitate early diagnosis and initiate timely treatment to prevent irreversible vision loss.
